# Five Questions about Microsporidia

**DOI:** 10.1371/journal.ppat.1000489

**Published:** 2009-09-25

**Authors:** Patrick Keeling

**Affiliations:** Canadian Institute for Advanced Research, Botany Department, University of British Columbia, Vancouver, British Columbia, Canada; University of California San Francisco, United States of America

## What Are Microsporidia?

**Figure 1:**
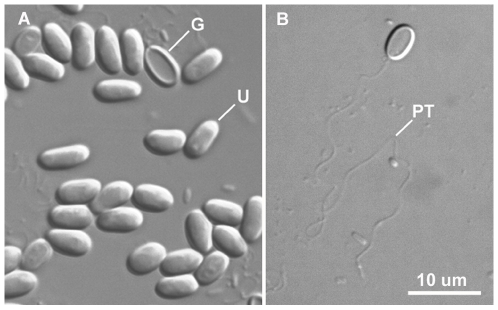
Light micrograph of *Antonospora locustae* with pressure-induced polar tube eversion. The scale bar is 10 um. (A) Many ungerminated spores (one example labeled U) and a few germinated spores, showing the residual spore wall (one example labeled G). (B) A germinated spore where the everted polar tube (PT) has extended far from the cell and can be seen to be many times the length of the spore.

Microsporidia are a diverse group of obligate intracellular eukaryotic parasites. There are approximately 1,300 formally described species in 160 genera [1], but this certainly represents a tiny fraction of the real diversity because most potential host lineages have been poorly surveyed. Nearly all microsporidia are known to infect animals, and some are responsible for a number of human diseases (13 species of microsporidia have been documented to infect humans) predominantly associated with immune suppression [2]. They also infect several commercially important animal species such as bees, silk worms, and salmon, and various domesticated mammals. They are thought to be especially common in insects and fish, although most invertebrates have been so poorly surveyed this may change. Their infective stage is a thick-walled spore, which is also the only stage that can survive outside their host cell [3]. The spore contains a sophisticated infection apparatus, primarily distinguished by a long, coiled filament called the polar filament. When the spore germinates, an inflow of water leads to pressure in the spore that eventually ruptures the wall and forces the polar filament to eject, turning inside out to form a tube (Figure 1) [4]. This process takes place very quickly, so the polar tube is in effect a projectile. At the completion of germination, the parasite cytoplasm is forced through the tube and either delivered to the surface of the host cell, or perhaps injected into the host cytoplasm if the projectile tube has actually penetrated the host cell. It has also been shown that microsporidia can be taken up by phagocytosis, and then use the polar tube to escape from the vacuole [5], so there appear to be more than one mode of infection.

## Are They Protists, Fungi, or What?

**Figure 2:**
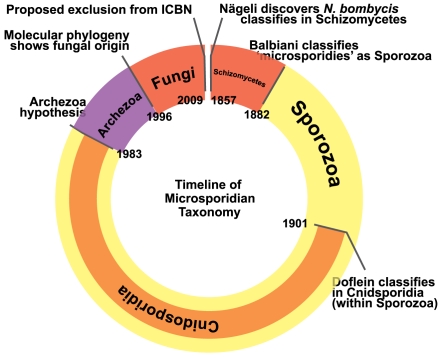
Timeline of the changing taxonomic position of microsporidia, from their discovery in 1857 to the present. When first described in 1857, they were classified as schizomycete fungi. Later they were consisdered sporozoan protists (and more specifically members of the subgroup Cnidosporidia), a position favoured for over 100 years. In 1983 a new hypothesis radically departed from this idea, suggesting they were an ancient, primitive lineage that evolved before the origin of mitochondria. Molecular data originally supported this possibility, but as data accumulated, it became clear that they were in reality highly reduced fungi, a conclusion broadly supported by the genomic data now available, although their exact relationship to fungi remains contentious.

There has been considerable debate about the origin of microsporidian parasites. Aside from their elaborate infection mechanism, they have few distinguishing features, and have thus been difficult to compare to other eukaryotes. This is illustrated by their tumultuous taxonomic history (Figure 2), and the tendency to lump them with what we now know to be unrelated organisms. When microsporidia were discovered in 1857, they were considered to be schizomycete fungi, but this was an artificial group that included various yeasts and bacteria. They were soon transferred to Sporozoa and ultimately to the subgroup Cnidosporidia. This too was a grab-bag of four unrelated groups (Microsporidia, Myxosporidia, Actinosporidia, and Helicosporidia) that were falsely grouped because of their intracellular parasitic way of life. Remarkably, Microsporidia, Myxosporidia, and Helicosporidia have since been shown to be fungi, animals, and green algae, respectively, underscoring just how distantly related these parasites really are. Eventually the absence of many “eukaryotic” features in microsporidia, in particular mitochondria, led to the proposal that microsporidia never had these features because they diverged from other eukaryotes prior to the origin of these features [6]. Microsporidia and a few other lineages without obvious mitochondria were collectively called “Archezoa” and were thus proposed to be ancient, primitive lineages of great importance to understanding the origin of eukaryotes. Molecular data originally supported this “ancient origin” hypothesis [7], but as the sampling of genes increased, another hypothesis emerged: that microsporidia are related to fungi [8],[9]. Since the 1990s, most well-supported gene trees have shown this fungal connection [10], and the support for trees that showed the deep-branching position has been undermined by analysis with more sophisticated models that take into account rate heterogeneity [11],[12]. The completion of the *Encephalitozoon cuniculi* genome underlined this transition in our thinking [13], and also revealed the correlation between substitution rate and “deep-branching” [14]. The conclusion that microsporidia are fungi has one unwanted implication: all taxa that were named based on the view that they are protists (about 1,000 names) are invalid because fungi are subject to botanical rules of nomenclature. Accordingly, it has now been proposed that microsporidia are excluded from the International Code of Botanical Nomenclature, despite their fungal nature [15]. Our view of the origin of microsporidia has thus come full circle, in a way. Their original classification as fungi was actually based on a misguided view of microbial diversity, but we have nonetheless returned to the view that microsporidia are fungi, although where they might branch in relation to the various fungal phyla has remained a source of debate [16].

## Are They Really “Amitochondriate”?

The Archezoa hypothesis (that microsporidia were an ancient, primitive lineage) was based on the absence of mitochondria in microscopy studies [6]. However, the phylogenetic evidence that microsporidia are closely related to fungi made it impossible for them to have been ancestrally amitochondriate, thus begging the following question: do they still have mitochondria, or did they lose them? The first evidence for mitochondrial relicts came in the form of nuclear-encoded genes for mitochondrion-targeted proteins. The first of these to be found was HSP70, followed by pyruvate dehydrogenase [17]–[20], and eventually a handful of other genes in the complete genome of *E. cuniculi* [13]. These confirmed the mitochondriate ancestry of the microsporidia, but left some room for doubt about whether a relict organelle actually persisted because the evidence that these genes encoded organelle-targeting transit peptides was far from clear-cut. Because of this, even the complete genome of *E. cuniculi* could only provide indirect support for the presence of an organelle in the cell. Direct evidence for the retention of mitochondria came from the immuno-localisation of HSP70 in *Trachipleistophora hominis*, which reveled multiple, tiny (50×90 nm) organelles bounded by two membranes, but lacking any other distinguishing structural features [21]. This derived and reduced mitochondrion was named a mitosome. Subsequently, several genes involved in the assembly of iron-sulfur clusters were localized to the mitosomes of *E. cuniculi* and *T. hominis* [22],[23]. Interestingly, however, not all mitochondrion-derived proteins still function in the mitosome: the *E. cuniculi*, glycerol-3-phosphate dehydrogenase, and some components of iron-sulfur cluster assembly in *T. hominis* localize to the cytosol, despite their mitochondrial ancestry [22],[23]. These proteins have therefore found a new cytosolic function as the organelle degenerated, suggesting the functions of the organelle are even more limited than the genomic data led us to believe.

## What Are Microsporidian Genomes Like?

Microsporidian genomes are made up of multiple linear chromosomes much like that of other eukaryotes, but they are otherwise quite reduced and unusual. For a start, many microsporidian genomes are quite small. At the extreme, the *Encephalitozoon intestinalis* genome is only 2.3 Mbp, smaller than many bacterial genomes. The complete genome of *E. cuniculi* is only 2.9 Mbp, and only encodes about 2,000 protein-coding genes [13]. The genome is highly compacted, with short intergenic regions, almost no repeats, and little evidence of selfish elements, altogether leading to a gene density approximately twice that of *Saccharomyces*. Even the genes themselves are shorter than homologues in other fungi, a likely consequence of domain loss due to the reduction of interaction networks. *E. cuniculi* genes have also massively reduced the number of introns they contain: only 14 have been annotated at present in the entire genome, and in *Enterocytozoon bieneusi* they appear to have been eliminated altogether [24]. The close proximity of the genes to one another in these compact genomes seems to have an effect on transcription, since a high frequency of overlapping transcripts has been observed in both *E. cuniculi* and *Antonospora locustae* [25],[26]. In particular, transcription termination is often not immediately after a gene, but well into or beyond the adjacent gene, so that an mRNA can contain sequence from more than one gene, although only one gene appears to be translated (as often as not, the additional genes are in the opposite strand). This makes it difficult to interpret large scale transcription patterns, since the presence of RNA corresponding to a gene does not necessarily mean that gene is being expressed. The genomes of these highly compacted species have attracted the most attention, but we are now beginning to see that other microsporidian genomes are quite different. There is a nearly a 10-fold range in genome size between different species that have been investigated [27], and genomes on the larger side of the spectrum have been shown to have a low gene density and many transposons [28],[29].

## How Do Microsporidia Depend On Their Host?

Microsporidia cannot grow or divide outside their host cell, but exactly how they interact and use resources from their host is only partially known. It has long been known that infection induces changes in the host that appear to be related to metabolic dependency. For example, infection by several species leads the host to surround the parasite with mitochondria, presumably supplying the parasite with energy. In one extreme case, infection leads the host cell to grow to an enormous, mutinucleate cell called a xenoma, which becomes a highly organized spore production assembly line [30]. Prior to the widespread use of genomic methods, the metabolism of microsporidia was difficult to study because they were difficult to separate from their host cells, but it has long been clear they lack many metabolic pathways, such as oxidative phosporylation, electron transport, and tricarboxylic acid cycle [31]. Characterisation of genes involved in metabolism, and in particular genome surveys of microsporidia, have confirmed and refined this view. In all well-sampled species there are no genes for many metabolic pathways (e.g., tricarboxylic acid cycle), and few genes relating to the synthesis of small molecules such as amino acids and nucleotides. Interestingly, several ATP transporters have been found in microsporidia. Some of these localize to the outer membrane of the parasite and seem to import ATP from the host cell, while others import ATP into the relict mitosome [32],[33]. There is also some variability in the metabolic capacity of different species of microsporidia, the most extreme case being the human parasite *E. bieneusi*, with a genome sequence survey revealing virtually no genes for central carbon metabolic pathways [24]. This includes glycolysis, which is the backbone of energy generation in other microsporidia, suggesting that this species is unable to generate energy from sugar, and is therefore dependent on its host directly for ATP, making this species one of the most host-dependent parasites known.
